# Development and characterization of canine‐specific computational models to predict pulsatile arterial hemodynamics and ventricular‐arterial coupling

**DOI:** 10.14814/phy2.15731

**Published:** 2023-06-03

**Authors:** Julia C. Hotek, Julio A. Chirinos, Theodore J. Detwiler, Hillary K. Regan, Christopher P. Regan

**Affiliations:** ^1^ Preclinical Development (PCD) Merck & Co., Inc. West Point Pennsylvania USA; ^2^ Perelman School of Medicine University of Pennsylvania Philadelphia Pennsylvania USA

**Keywords:** aortic pressure waveform, hemodynamics, pulse wave analysis, safety pharmacology, telemetry, wave power analysis, wave separation analysis

## Abstract

Pulsatile hemodynamics analyses provide important information about the ventricular‐arterial system which cannot be inferred by standard blood pressure measurements. Pulse wave analysis (PWA), wave separation analysis (WSA), and wave power analysis (WPA) characterize arterial hemodynamics with limited preclinical applications. Integrating these tools into preclinical testing may enhance understanding of disease or therapeutic effects on cardiovascular function. We used a canine rapid ventricular pacing (RVP) heart failure model to: (1) Characterize hemodynamics in response to RVP and (2) assess analyses from flow waveforms synthesized from pressure compared to those derived from measured flow. Female canines (*n* = 7) were instrumented with thoracic aortic pressure transducers, ventricular pacing leads, and an ascending aortic flow probe. Data were collected at baseline, 1 week, and 1 month after RVP onset. RVP progressively reduced stroke volume (SV), the PWA SV estimator, and WSA and WPA pulsatility and wave reflection indices. Indices derived from synthesized flow exhibited similar directional changes and high concordance with measured flow calculations. Our data demonstrate the value of analytical hemodynamic methods to gain deeper insight into cardiovascular function in preclinical models. These approaches can provide complementary value to standard endpoints in evaluating potential effects of pharmaceutical agents intended for human use.

## 1 INTRODUCTION

Changes in cardiovascular (CV) function, primarily determined by aortic blood pressure measurement, are important variables in demonstrating the potential for clinical CV events (McEniery et al., 2014) and determination of the risk for adverse events in drug development. Undesired CV effects are a major cause of drug attrition during preclinical and clinical development. Therefore, blood pressure is a primary parameter collected in CV safety pharmacology studies (Bhatt et al., 2019; Laverty et al., 2011; U.S. Department of Health and Human Services Food and Drug Administration, 2001). Typically, changes in blood pressure are reported as deviations in systolic, diastolic, and derived mean pressures. While valuable, directional changes in these endpoints offer minimal mechanistic insight, such that any effects on blood pressure often necessitate additional studies in models requiring complex chronic instrumentation (Vasan et al., 2001). More importantly, it has become clear that standard blood pressure measurements can be insensitive to important hemodynamic phenotypes that affect the risk of cardiovascular disease in humans, and could therefore miss clinically‐relevant hemodynamic effects in preclinical testing.

Aortic pressure waveforms contain valuable information that can be used to characterize pulsatile hemodynamics and ventricular‐arterial interactions beyond standard blood pressure indices (McEniery et al., 2014), therefore providing insights into arterial and/or cardiac function and into pharmacologic effects of novel drugs in the setting of preclinical drug development.

Analytic approaches have been extensively used in human clinical research studies to analyze arterial pressure waveforms to gain further mechanistic information regarding central hemodynamics (Chirinos & Segers, 2010; Denardo et al., 2010; Hashimoto et al., 2008; Kondiboyina et al., 2023; Paglia et al., 2014; Townsend et al., 2015; Weber et al., 2012; Weber & Chirinos, 2018), providing insights about pulsatile load, ventricular function, and ventricular‐arterial interactions (Adji et al., 2011; Chirinos, 2013; Chirinos & Segers, 2010; Denardo et al., 2010; Hametner et al., 2017; Wilkinson et al., 2002). Common analytical methods include pulse wave analysis (PWA), wave separation analysis (WSA), and wave power analysis (WPA), an extension of wave intensity analysis (WIA) to volume flow (rather than flow velocity) measurements. Integrating these tools into preclinical safety or efficacy testing may enable greater understanding of disease or effects of novel therapeutic agents on heart and vascular function. However, applications of these methods in animals have been very limited.

In this study, we used a well‐characterized canine rapid ventricular pacing (RVP) induced heart failure model to accomplish the following aims: (1) to characterize pulsatile hemodynamic changes in response to RVP, an intervention that primarily induces a cardiomyopathic response; (2) to assess the performance of pressure‐flow analyses using flow waveforms synthesized from the pressure waveform compared to those derived from measured flow.

## 2 METHODS

### 2.1 Study design

All animal studies were conducted in accordance with the Guide for the Care and Use of Laboratory Animals (Institute of Laboratory Animal Resources, Commission on Life Sciences, National Research Council, 2011) and were approved by the Institutional Animal Care and Use Committee at Merck & Co., Inc. Calibrated waveforms for all studies were collected using the Notocord data acquisition system (HEM version 4.3.0.67 or later).

### 2.2 Pacing‐induced cardiac dysfunction

Briefly, mixed breed female canines (*n* = 7; 17–20 kg, Marshall Bioresources) were anesthetized with propofol (2–3 mg/kg, iv), and maintained via inhaled isoflurane (1%–2%) in oxygen. A thoracotomy at the fourth–fifth intercostal space was performed and solid‐state pressure transducers connected to an implantable telemetry device (model TF27G, ITS, Konigsberg Instruments) were placed in the aortic arch just distal to the carotid bifurcation and in the left ventricle to record arterial pressure and left ventricular pressure, respectively. The telemetry module was secured intramuscularly in the left flank. A flow probe (Transonics, Inc.) was placed around the ascending aorta proximal to the brachiocephalic bifurcation and secured in placed to record aortic flow. Additionally, two epicardial pacing electrodes were placed on the right ventricle. Flow probe cables and wires from the pacing electrodes terminated in titanium skin buttons secured dorsally between the scapulae. Analgesics were administered pre‐ and post surgery and animals were under the care of attending veterinary staff. Animals were allowed at least 4 weeks recovery prior to the start of studies.

Animals were trained to stand in a laboratory sling for at least 1 h with stable CV parameters. Following surgical recovery, basal hemodynamics was assessed in sling‐trained conscious dogs prior to the start of pacing (noted as “baseline”) and during the development and progression of cardiac dysfunction (5–7 days after pacing onset noted as “1 week”, 21–31 days noted as “1 month”) (Regan et al., 2016). During each recording session, 5 min of continuous waveform data collected at 500 Hz for each animal was extracted for analysis. Rapid ventricular pacing (RVP) at a rate of 240 bpm was initiated after baseline measurements, then reduced to rates ranging from 200 to 240 bpm based on clinical and hemodynamic assessment on Day 11 or later. Animals were checked daily to ensure chronic pacing and animal health. Hemodynamics and cardiac function were assessed at baseline, 1 week, and 1 month after pacing onset. To assess hemodynamics after initiation of RVP, pacing was stopped approximately 30–45 min prior to the start of data collection to allow for equilibration. Stroke volume (SV) was calculated directly from aortic flow in Notocord.

### 2.3 Pulse wave analysis

All PWA signal processing was performed in Matlab R2021a (MathWorks). Blood pressure signals were filtered with a Savitzky–Golay smoothing filter having a window length of 23 and a polynomial order of 2 and low‐pass filtered with a cutoff frequency of 10 Hz (Peltokangas et al., 2017). Systolic and diastolic peaks were identified by detecting the peak and nadir of each pressure wave. Signals were filtered based on moving averages of 10 samples to eliminate erroneous peaks.

**FIGURE 1:**
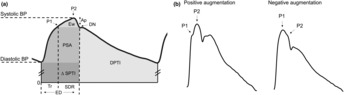
(a) Example aortic blood pressure waveform and pulse wave analysis (PWA) indices. P1 represents the first shoulder or early systolic peak, which in this case is the inflection point of the forward and reflected pressure waves. P2 represents the late systolic peak, which is also the systolic peak of this waveform. DN, or the dicrotic notch, is the incisura that marks the end of systole. PWA pressure augmentation, time intervals, and areas under the curve can be computed from these parameters and the diastolic pressure. Augmented pressure (Ap) is the amplitude difference between the early (P1) and late (P2) systolic peaks and is a measurement of the intensity of the reflected wave (Nichols et al., 2015). Ejection duration (ED) measures the time of systole, from the upstroke of the pressure wave at the diastolic nadir to the DN, and is composed of Tr and SDR (Nichols et al., 2013). The round trip travel time (Tr) to and from the major reflection sites of the body is measured from the diastolic pressure to the early inflection point (P1), while systolic duration of the reflected wave (SDR) is the time from *P*1 to DN (Hashimoto et al., 2008). The area under the systolic portion of the reflected wave above P1 is the LV wasted effort (*E*_w_) (Hashimoto et al., 2008), while the area under the systolic portion of the pressure curve above diastole is the pressure systolic area (PSA) (Denardo et al., 2010; Kouchoukos et al., 1970). The systolic pressure time integral (SPTI) is the total area under the systolic portion of the pressure wave (SPTI = ΔSPTI+PSA + *E*_w_) and the diastolic pressure time integral (DPTI) is the area under the diastolic portion of the pressure wave (Nichols et al., 2013). (b) Representative waveforms illustrating P1 and P2 identification in the cases of positive and negative augmentation.

A signal‐averaging algorithm determined the pressure wave augmentation and the location of the first shoulder (*P*1), late systolic peak (*P*2), and dicrotic notch (DN) in blood pressure waveforms based on higher order derivatives (Figure 1a) (Hayward & Kelly, 1997). Briefly, groups of eight consecutive waveforms are normalized in amplitude and dicrotic notch duration and signal‐averaged to obtain a mean normalized waveform for that nth group. For the nth mean normalized pressure waveform $f_{n}$, the first through fifth derivatives ($f_{n}^{\prime }$,$\;f_{n}^{\prime \prime },f_{n}^{\prime \prime \prime },f_{n}^{\left(4\right)},f_{n}^{\left(5\right)}$) are computed, approximated by finite differences and with respect to time. The nth waveform is analyzed based on the sign of $f_{n}^{\left(5\right)}$ at the point corresponding to peak pressure (Peltokangas et al., 2017) (Figure 1b). In the case of positive augmentation, where $f_{n}^{\left(5\right)}$ is negative, the maximum blood pressure is the late systolic peak, *P*2 (Hayward & Kelly, 1997). The second zero crossing of $f_{n}^{\left(4\right)}$ from positive to negative after the maximum $f_{n}^{\prime }\;$is then assigned to the inflection point *P*1 (Katsuda et al., 2013; Kelly et al., 1989). In the case of negative augmentation, where $f_{n}^{\left(5\right)}$ is positive, the maximum blood pressure is assigned to P1. The inflection point P2 is the first zero crossing of $f_{n}^{\left(4\right)}\;$from negative to positive after P1. In all cases, DN is identified as the first zero‐crossing of $f_{n}^{\prime \prime \prime }$ from positive to negative after the maximum blood pressure. P1, P2, and DN positioning on $f_{n}$ is then used to locate these indices on the 8 waveforms in the nth group.

Wasted energy (*E*_w_) is calculated as the area under the systolic portion of the reflected wave (Figure 1a), or the area above the inflection point, *P*1 or *P*2 as determined by waveform augmentation (Hayward & Kelly, 1997). Pressure systolic area (PSA) is then calculated as the area under the blood pressure waveform left of the DN above diastolic blood pressure minus *E*_w_ (Figure 1a).

### 2.4 WSA and WPA

**FIGURE 2:**
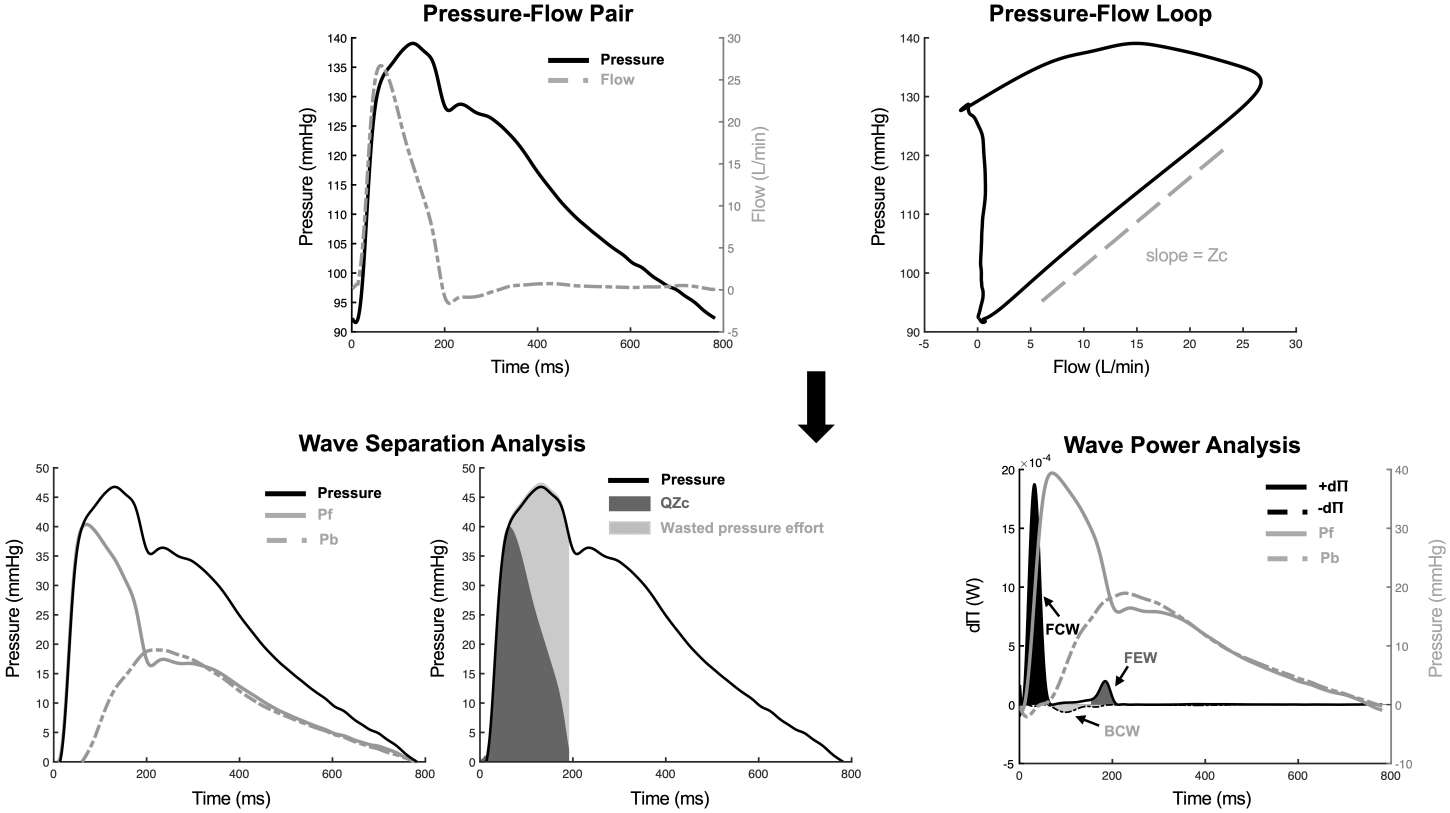
Example simultaneous aortic pressure and flow waveforms and wave separation analysis (WSA) and wave power analysis (WPA) indices. Aortic pressure and flow waveforms are acquired, and the slope of the early systolic pressure‐flow loop is approximated to equal the aortic characteristic impedance Zc. In WSA, aortic pressure is scaled to its diastolic pressure and separated into forward (Pf) and backward (Pb) pressure (Chirinos & Segers, 2010). Flow is multiplied by aortic Zc to obtain the product QZc, and the difference between QZc and measured pressure is quantified as Wasted pressure effort (Chirinos, 2017). In WPA, changes in pressure (dP) and flow velocity (dQ) are multiplied to calculate instantaneous wave power (d∏). Waves are identified as forward (d∏+) or backward (d∏−) based on direction of power peaks. These waves are further characterized as compression or expansion wavefronts based on signs of dP and dU: Forward compression wave (FCW): +d∏, + dP, +dQ; forward expansion wave (FEW): +d∏, −dP, −dQ; backward compression wave (BCW): −d∏, +dP, −dQ (Mynard & Smolich, 2016).

All WSA and WPA signal processing were performed in Matlab R2021a (MathWorks; Natick, MA). After signal averaging of pressure and flow waveforms, simultaneous aortic blood pressure (*P*) and aortic flow (*Q*) are used to decompose pressure into its forward (Pf) and backward (Pb) components. In this method, the slope of the early systolic pressure‐flow relationship (Figure 2) is calculated. This represents the proximal aortic characteristic impedance (Zc). Pf and Pb (Figure 2) measured as functions of time are determined via the following equations (Chirinos & Segers, 2010):

$\mathrm{Pf}=\frac{P+Q\times \mathrm{Zc}}{2}$ (1)

$\mathrm{Pb}=\frac{P-Q\times \mathrm{Zc}}{2}$ (2)

For each pair of generated Pf and Pb waveforms, Pf and Pb amplitudes are calculated as the difference between maximum and minimum pressures. A single value for reflection magnitude (RM), a parameter used to characterize wave reflections, is then calculated as the ratio of the backward and forward wave amplitudes (Pb/Pf).

Reflected waves are re‐reflected once they reach the left ventricle (LV), thus contributing to the forward wave (Phan et al., 2016). Therefore, RM is a problematic marker of wave reflection, given that reflected waves contribute to both its numerator and its denominator. Moreover, the ratio of amplitudes of Pb and Pf do not account for the adverse effect of wave reflections arriving at the aorta during systole (which increase LV mid‐to‐late systolic load) relative to the salutary effect of reflections arriving in diastole (when they do not increase LV afterload, but rather promote coronary perfusion). Therefore, additional useful indices that inform on the impact of wave reflection on pulsatile pressure‐flow relationships and ventricular‐arterial coupling can be determined from P, Q, and Zc, as previously described (Chirinos & Sweitzer, 2017): (1) the time integral of the product of flow and aortic Zc (QZc), which can be defined as the pulsatile pressure integral required to push the pulse flow waveform through the aortic root Zc; (2) Wasted LV effort, which is the difference between measured pressure and QZc and represents the additional pressure–time integral necessary to overcome wave reflections (Figure 2) (Chirinos, 2017). The ratio between wasted LV effort and QZc (wasted effort/QZc) was also computed. This is a dimensionless index analogous to RM, but it is based on systolic pressure–time integrals rather than amplitudes, and accounts for both magnitude and timing of wave reflections relative to LV ejection.

WPA was used as an alternative to WIA, as we measured volume flow rather than flow velocity (Mynard & Smolich, 2016). In WPA, instantaneous rates of change of blood pressure (dP) and flow volume (dQ) are computed, and wave power (d∏) is calculated as the product of the measured dP and dQ (Figure 2) (Mynard & Smolich, 2016; Weber & Chirinos, 2018). Forward waves are identified by positive power peaks (+d∏), while backward waves are identified by negative power peaks (−d∏). These waves are further defined as compression or expansion (decompression) waves based on the signs of dP and dQ (Forward compression wave (FCW): +d∏, +dP, +dQ; Forward expansion wave (FEW): +d∏, −dP, −dQ; Backward compression wave (BCW): −d∏, +dP, −dQ) (Mynard & Smolich, 2016). The height of FCW and FEW peaks were measured for further analysis in this study.

### 2.5 Determination of synthetic flow from aortic blood pressure

We also assessed the value of synthesizing the flow from blood pressure waveforms to determine whether computationally‐synthesized flow waveforms can eliminate the need for measured aortic flow, which required additional instrumentation, in WSA and WPA methods. WSA and WPA indices were determined using personalized synthetic flow waveforms derived from custom software written in Matlab (MathWorks), as previously published (Shenouda et al., 2021).

In this approach, a “personalized” flow waveform was synthesized from each aortic blood pressure waveform using an algorithm designed by Chirinos and validated in humans by Shenouda et al. (2021) and Armstrong et al. (2022). Briefly, this approach is based on well‐established hemodynamic principles, including: (1) pressure and flow waveforms increase together and are concordant with each other until approximately the time of the inflection point in the pressure waveform; (2) the late systolic portion of the pressure and flow waveforms decay in parallel in late systole as a result of a forward‐traveling suction wavefront (decompression or expansion wave) generated by the end of contraction and initiation of left ventricular relaxation; (3) for both of these phenomena, the pressure‐flow relation is governed by aortic Zc; (4) a lower frequency reflected wave with a quasi‐linear upstroke adds to forward pressure in mid‐to‐late systole; and (5) flow scaling (and thus the absolute value of Zc) are not important for wave separation analysis. In previous studies, this algorithm was applied until the end of LV ejection (dicrotic notch), with flow values assuming to equal zero values thereafter, to synthesize a flow waveform similar to that measured at the LV outflow tract with echocardiography, in which no diastolic flow normally occurs. However, as aortic flow profiles are not flat in early diastole, we extended the synthetic process until the point of maximum diastolic pressure after the dicrotic notch, which effectively reproduced the brief diastolic flow reversal observed in the aorta, based on the pressure waveform morphology in the vicinity of the dicrotic notch (Figure 2).

### 2.6 Statistical analyses

Various physiologic parameters and raw SV or PSA were presented as the average values calculated over 5 min of consecutive data. For each parameter, the effects of pacing were examined by comparing each measure at different timepoints using a one‐way repeated‐measures analysis of variance (ANOVA), followed by post hoc multiple comparisons with the Bonferroni correction. To directly compare RVP‐induced changes in each parameter, SV and PSA data at 1 week and 1 month were normalized to baseline values for each animal. Baseline‐normalized SV and PSA were compared directly at each time point (SV vs. PSA) and across time (1 week vs. 1 month) using a two‐way repeated‐measures ANOVA, followed by post hoc multiple comparisons with the Bonferroni correction when overall differences were detected. Association between baseline‐normalized PSA and SV was evaluated using Spearman's rho coefficient.

WSA and WPA parameters were derived from blood pressure and true flow or synthetic flow data and presented as the average values calculated over 5 min of data. For each parameter, a two‐way repeated‐measures ANOVA examined the effects of time and flow type used in parameter generation (true flow vs. synthetic flow), followed by post hoc multiple comparisons with the Bonferroni correction. In the cases where synthetic flow values were not generated (Zc, FCW height, FEW height), a one‐way repeated measures ANOVA was performed to evaluate the effects of pacing on true flow‐derived values, followed by post hoc multiple comparisons with the Bonferroni correction when overall differences were detected.

Association between measured flow and synthetic flow derived indices was further evaluated using Spearman's rho correlation. Lastly, the agreement in measured flow and synthetic flow derived indices was assessed using Bland–Altman plots, through the assessment of mean bias and the range of the 95% LOA.

Level of statistical significance was set equal to 0.05, and data are expressed as means ± standard deviation. Statistical analyses were performed using R Statistical Software (version 4.0.1; R Foundation for Statistical Computing).

## 3 RESULTS

### 3.1 Pulse wave analysis

**FIGURE 3:**
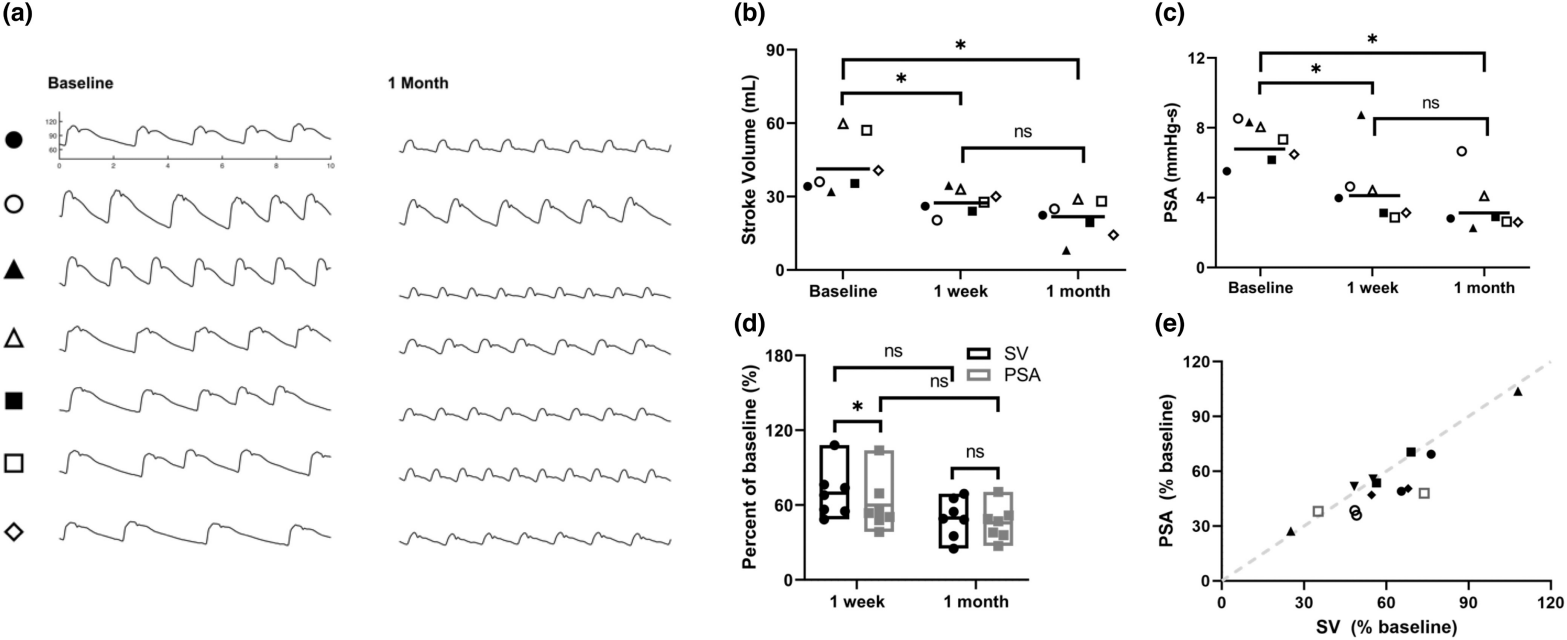
(a) 10 s of representative data for each animal at baseline and 1 month time points. Symbol on left corresponds to animal‐identifying symbols used in panels B‐G. Y‐axis for all data segments is set at 40–140 mmHg. (b) Average stroke volume (SV) measurement taken over a 5 minute recording period for each animal at baseline, 1 week, and 1 month. Data from individual animals are indicated by symbol. Horizontal lines represent mean value at each time point. *p < 0.05. (c) Average pressure systolic area (PSA) measurement taken over a 5 min recording period for each animal. (d) SV and PSA data at 1 week and 1 month normalized to baseline data for each animal. Bars represent minimum to maximum data, with a horizontal line at the mean value. (e) Comparison between SV and PSA reported as percent of baseline measurements. The dashed line represents equality.

RVP‐induced changes on various physiologic parameters (Table 1; Figure 3a) were assessed. RVP progressively reduced both SV (1 week: 69.5 ± 19.9%, *p* = 0.044; 1 month: 49.6 ± 15.6%, *p* = 0.001) and PSA (1 week: 60.7 ± 21.9%, *p* = 0.014; 1 month: 47.1 ± 16.1%, *p* < 0.001) compared to baseline (Figure 3b–d). There was no significant difference between 1 week and 1 month data for either variable (*p* > 0.38; Figure 3b–d). SV and PSA reductions from baseline were different at 1 week (*p* = 0.035; Figure 3d) but were not significantly different from each other at 1 month (*p* = 0.264). A scatterplot of SV against PSA is shown in Figure 3e. There was a strong positive correlation between the percent change from baseline for SV and that of PSA (Spearman's rho = 0.745; *p* = 0.003; Figure 3e).

**TABLE 1:** Progression of changes in various hemodynamic parameters and PWA data at baseline and 1 week and 1 month after start of continuous RVP.

	Baseline	1 week	1 month
Heart rate (bpm)	86.5 ± 9.7	99.1 ± 18.5	120.9 ± 23.1*
Mean arterial pressure (mmHg)	92.1 ± 11.5	71.1 ± 7.8*	70.7 ± 10.8*
Left ventricular end diastolic pressure (mmHg)	9.1 ± 1.5	11.8 ± 6.6	18.2 ± 4.1*
+dPdt (mmHg/s)	3313 ± 471	1597 ± 539*	1359 ± 156*
Stroke volume (mL)	41.2 ± 11.5	28.0 ± 5.0*	20.8 ± 7.6*
Pressure systolic area (mmHg‐s)	6.8 ± 1.1	4.1 ± 2.0*	3.1 ± 1.2*

*Note*: Data are expressed as mean ± standard deviation.

Abbreviations: PWA, pulse wave analysis; RVP, rapid ventricular pacing.

* *p* < 0.05 versus baseline values.

### 3.2 WSA and WPA

**FIGURE 4:**
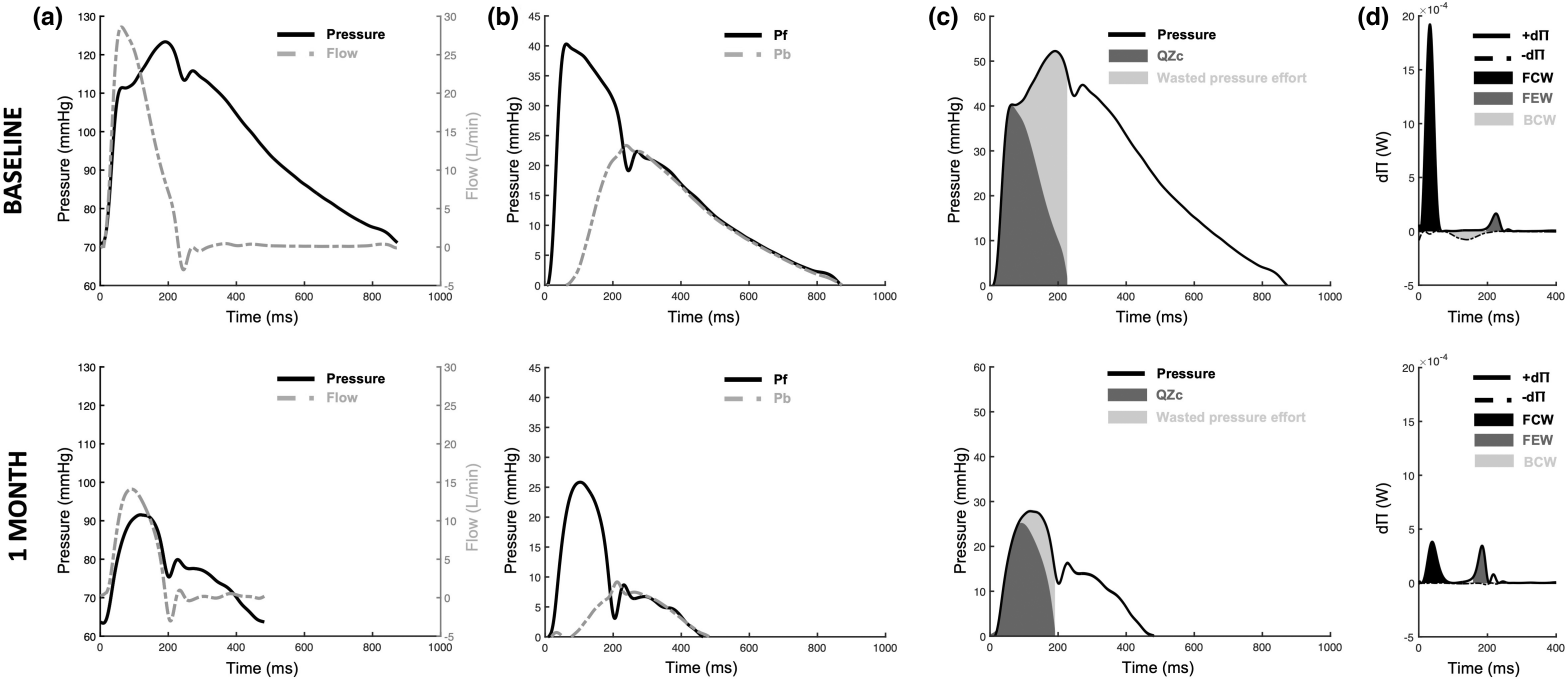
Representative aortic blood pressure, flow, and wave separation analysis (WSA) and wave power analysis (WPA) from animal #4 at baseline (top) and 1 month after onset of RVP (bottom). (a) Simultaneous pressure (solid black line) and flow (dashed gray line) waveforms were collected. (b) Aortic pressure is scaled by setting the diastolic blood pressure to zero and then separated into forward (Pf, solid black line) and backward (Pb, dashed gray line) pressure waves, where reflection magnitude (RM) is the ratio between the peaks of Pb/Pf. (c) After Zc is determined from pressure‐flow loops, the QZc product (dark gray area) is identified and wasted pressure effort (light gray area) is then calculated as the difference between QZc and measured pressure (solid black line). (d) Change in flow volume (dQ) and pressure (dP) are used to determine forward (+d∏, solid black line) and backward (−d∏, dashed black line) wave power. The signs of dQ, dP, and d∏ classify waves as forward compressive (FCW, black area), forward expansion (FCW, dark gray area), and backward compression (BCW, light gray area) waves.

WSA and WPA methods were applied to simultaneous aortic blood pressure and measured aortic flow data (Table 2, Figure 4a). RVP significantly reduced forward (Pf; 1 week: *p* = 0.002; 1 month: *p* = 0.001) and backward (Pb; 1 week: *p* = 0.008; 1 month: *p* = 0.004) pressure wave amplitudes and reflection magnitude (RM), which is the ratio between the two wave reflection amplitudes (Pb/Pf; 1 week: *p* = 0.016; 1 month: *p* = 0.049; Figure 4b). RVP had no effect on aortic Zc (*p* = 0.398; Table 2) but significantly reduced the flow Zc product (QZc; 1 week: *p* = 0.012; 1 month: *p* = 0.002), wasted effort (1 week: *p* = 0.004; 1 month: *p* < 0.001), and the wasted effort/QZc ratio (1 week: *p* = 0.003; 1 month: *p* < 0.001; Figure 4c) at both time points.

**TABLE 2:** Progression of changes in various WSA and WPA parameters at baseline and 1 week and 1 month after start of continuous RVP, calculated using aortic blood pressure and measured aortic flow data (left) or synthetic flow data (right).

	Measured flow			Synthetic flow		
	Baseline	1 week	1 month	Baseline	1 week	1 month
Forward pressure wave (Pf) amplitude (mmHg)	42.3 ± 7.0	29.6 ± 11.1*	25.4 ± 7.4*	44.6 ± 8.7***	31.9 ± 13.2* ^,^ ***	23.1 ± 6.0*
Backward pressure wave (Pb) amplitude (mmHg)	20.3 ± 2.6	11.7 ± 5.4*	9.9 ± 5.2*	20.2 ± 2.5	10.9 ± 6.1* ^,^ ***	9.5 ± 5.7*
Reflection magnitude (RM)	0.48 ± 0.05	0.39 ± 0.05*	0.38 ± 0.089*	0.46 ± 0.06***	0.33 ± 0.04* ^,^ ***	0.40 ± 0.15
Characteristic impedance (Zc; mmHg‐min/L)	1.89 ± 0.52	1.95 ± 0.63	2.08 ± 0.73	–	–	–
Flow Zc product (QZc; mmHg‐ms)	4381 ± 748	3182 ± 1177*	2417 ± 893*	5053.2 ± 1187.4***	3489.3 ± 1746.6*	1937.3 ± 632.2* ^,^ ***
Wasted effort (mmHg‐ms)	3641 ± 494	1608 ± 1191*	1146 ± 813*	3079.8 ± 514.7***	1265.9 ± 622.0*	1626.8 ± 1254.6* ^,^ ***
Wasted effort/QZc	0.84 ± 0.11	0.47 ± 0.15*	0.45 ± 0.14*	0.64 ± 0.16^c^	0.37 ± 0.10*	0.85 ± 0.46** ^,^ ***
Forward compression wave (FCW) height (μW)	99.3 ± 33.5	30.3 ± 19.*	21.4 ± 7.3*	–	–	–
Forward expansion wave (FEW) height (μW)	12.1 ± 5.2	16.9 ± 4.8	16.6 ± 5.5	–	–	–
FCW/FEW height ratio	9.3 ± 2.8	2.0 ± 1.6*	1.3 ± 0.2*	8.8 ± 2.5	1.9 ± 1.2*	2.0 ± 1.0*

*Note*: Data are expressed as mean ± standard deviation.

Abbreviations: RVP, rapid ventricular pacing; WPA, wave power analysis; WSA, wave separation analysis.

* *p* < 0.05 versus baseline values

** *p* < 0.05 versus 1 week values

*** *p* < 0.05 versus time‐matched measured flow values.

The WPA parameters examining forward compression wave (FCW) and forward expansion wave (FEW) peak heights were assessed (Table 2; Figure 4d). RVP significantly reduced FCW height at 1 week and 1 month compared to baseline (1 week: *p* = 0.003; 1 month: *p* < 0.001), and there was no change in FEW height with pacing (*p* = 0.109). Therefore, the calculated ratio between FCW and FEW heights was significantly reduced at 1 week and 1 month following onset of RVP (1 week: *p =* 0.002; 1 month: *p* < 0.001).

### 3.3 Assessments using synthetic flow

**FIGURE 5:**
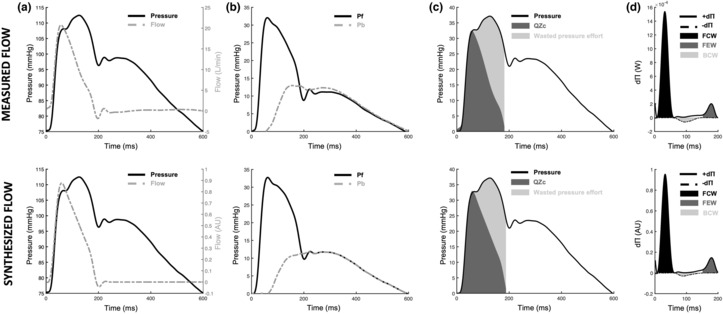
Representative aortic blood pressure, flow, and wave separation analysis (WSA) and wave power analysis (WPA) from animal #1 at baseline. The top row uses true measured flow measured from the animal, and the bottom row uses the synthesized flow waveform for WSA and WPA calculations. (a) Simultaneous pressure (solid black line) and measured flow (top) or synthesized flow (bottom) waveforms (dashed gray line) were collected/synthesized. (b) Aortic pressure is scaled by setting the diastolic blood pressure to zero and then separated into forward (Pf, solid black line) and backward (Pb, dashed gray line) pressure waves. (c) The QZc product (dark gray area) and wasted pressure effort (light gray area) for the corresponding measured pressure (solid black line). (d) Change in flow volume (dQ) and pressure (dP) are used to determine forward (+d∏, solid black line) and backward (−d∏, dashed black line) wave power. The signs of dQ, dP, and d∏ classify waves as forward compressive (FCW, black area), forward expansion (FCW, dark gray area), and backward compression (BCW, light gray area) waves. d∏ derived from synthesized flow (bottom trace) is uncalibrated, so FCW, FEW, and BCW heights are dimensionless but the ratio between these values can be determined.

To evaluate synthetic flow approaches, WSA and WPA parameters were also derived from synthesized flow waveforms at baseline, 1 week, and 1 month timepoints (Table 2; Figure 5a). In these analyses, similar to trends observed with measured flow, Pf (1 week: *p* = 0.003; 1 month: *p* < 0.001) and Pb (1 week: *p* = 0.012; 1 month: *p* = 0.002) were significantly reduced at 1 week and 1 month compared to baseline (Figure 5b). Reflection magnitude was different at 1 week (*p* = 0.017) but returned to baseline levels by 1 month (*p* = 1). Though Zc cannot be directly determined from synthetic flow as flow units are uncalibrated in the synthesized flow waveform, the product QZc can be evaluated as it is a pressure–time integral. Similar to trends observed with measured flow, QZc (1 week: *p* = 0.014; 1 month: *p* < 0.001) and wasted effort (1 week: *p* = 0.003; 1 month: *p* = 0.018) were significantly reduced at 1 week and 1 month after RVP compared to baseline (Figure 5c). In the case of synthetic flow, the wasted effort/QZc ratio was significantly reduced at 1 week following RVP onset (*p* = 0.009) but by 1 month increased to levels above baseline that were different from 1 week (*p* = 0.035) but not baseline (*p* = 0.31).

As synthetic flow is uncalibrated, FCW and FEW heights cannot be directly calculated with synthetic flow waveforms and compared to true flow‐derived values; however, the ratio of the heights FCW/FEW can be determined as it is a dimensionless quantity and independent of flow units (Table 2). Similar to what was observed with measured flow, the FCW/FEW height ratio was significantly reduced following RVP (1 week: *p* = 0.001; 1 month: *p* < 0.001; Figure 5d).

### 3.4 Correspondence of absolute values of indices derived from synthetic versus measured flow

Synthetic flow‐derived Pf, Pb, and RM were significantly different from time‐matched true flow values at 1 week, with only Pf and RM different from corresponding synthetic flow values at baseline (*p* < 0.046). Synthetic flow‐derived QZc, wasted effort, and wasted effort/QZc values were significantly different from corresponding true flow‐derived values at baseline and 1 month (*p* < 0.038). Finally, synthetic flow‐derived FCW/FEW height ratios were not different from true flow‐derived ratios at any timepoint (*p* > 0.06).

Bland‐Atlman plots were generated to calculate mean biases and 95% LOA between each index derived from measured flow versus the same index derived from synthetic flow. Mean biases and 95% LOA were calculated and the Bland–Altman plots are presented in Supplementary Material (Figure S1: QZc: −166.6 mmHg‐ms, −1574 to 1241 mmHg‐ms; wasted effort: 140.9 mmHg‐ms, −1234 to 1516 mmHg‐ms; wasted effort/QZc: −0.0321, −0.713 to 0.648; Pf: −0.755 mmHg, −7.165 to 5.656 mmHg; Pb: 0.446 mmHg, −0.953 to 1.844 mmHg; RM: 0.0199, −0.0923 to 0.132; FCW/FEW height ratio: −0.0911, −2.47 to 2.29). Additionally, there was a strong positive correlation between measured flow and synthetic flow derived values for QZc, wasted effort, Pf, Pb, RM, and FCW/FEW height ratio (*p* < 0.001 for all cases), while wasted effort/QZc exhibited a weaker correlation (*p* = 0.118). Correlation plots are presented in Supplemental Material (Figure S2).

## 4 DISCUSSION

In this study, we performed detailed longitudinal analyses of arterial pressure‐flow relations (PWA, WSA, and WPA) to characterize pulsatile arterial hemodynamics in a well‐established canine rapid ventricular pacing (RVP) induced heart failure model (Regan et al., 2016). We describe, for the first time, changes in pulsatile hemodynamics and ventricular‐arterial interactions in this cardiomyopathic model. We demonstrate pronounced changes in various indices of arterial function and ventricular‐arterial interactions 1 week and 1 month after RVP onset, compared to baseline. Given the need for complex instrumentation to acquire aortic flow, we also assessed the performance of analyses that use flow waveforms synthesized from the pressure waveform, compared to performance using measured flow. We found that indices derived from synthesized flow exhibited similar directional changes and general high concordance with indices derived from measured flow. Our study is the first to characterize arterial responses in a predominantly cardiomyopathic heart failure preclinical model and supports the validity of PWA algorithms to measure PSA from blood pressure, WSA and WPA for pressure‐flow analyses, and synthetic flow approaches for the assessment of multiple indices of pulsatile arterial hemodynamics. Through this, our data demonstrate the potential value of pulsatile hemodynamic algorithms as tools to provide additional mechanistic insight into preclinical CV‐dependent changes to complement standard blood pressure data without the need for additional complex instrumentation.

Various algorithms have been proposed and developed to estimate SV and/or cardiac output (CO = SV × HR) from arterial blood pressure through PWA, because SV and CO are critical parameters of cardiac function that require complex instrumentation and/or procedures that limit preclinical data collection options (i.e., chronic aortic flow probe implantation, sonomicrometer crystal implantation, acute cardiac catheterization, ultrasound‐derived measures). Though many studies have compared the effectiveness of these algorithms to determine the most reliable estimator, no single index has emerged as the superior algorithmic approach (Parlikar et al., 2007; Sun et al., 2005; 2009; Zhang et al., 2015). In this study, we chose to approximate SV with pressure systolic area (PSA), which is the area under the systolic portion of the pressure pulse above diastolic pressure (Figure 1a). PSA was selected as the indicator of changes in cardiac function in our canine model as it has previously been compared to features of poor SV performance in humans with heart failure (Denardo et al., 2010). While previous studies have used PSA to estimate CO and/or SV from central aortic pressure, many did not account for wasted energy (*E*_w_) from wave reflections in their PSA calculations (DeLoskey et al., 1978; Kouchoukos et al., 1970; Vakily et al., 2017; Verdouw et al., 1975); however, it is known that PSA without this *E*_w_ correction is only directly related to SV when *E*w is “small or zero” (Denardo et al., 2010). Thus, in this study PSA was determined to be the area under the systolic portion of the pressure curve minus *E*w (Figure 1a).

Ventricular‐arterial coupling assessment through WSA measurements (Table 2) highlights compensatory arterial mechanisms during the pathogenesis of heart failure in this model. The RVP‐induced model of heart failure is essentially cardiopathic: healthy canines with healthy hearts and vascular systems underwent weeks of pacing to induce sustained ventricular dysfunction (Table 1). We document important secondary arterial responses, which likely constitute adaptive mechanisms to reduce ventricular load. Interestingly, both QZc and wasted pressure effort decreased as the heart weakened in this purely cardiopathic model. The ratio between the two represents how inefficient the systolic pulse generation is and decreased with RVP; the arterial system thus adapted to behave more efficiently (Table 1), because no arterial dysfunction was induced with RVP. Further, characteristic impedance of arteries Zc did not change as this parameter is dependent on wall material stiffness and aortic root cross‐sectional area, which likely requires longer‐term remodeling (Table 2). A reduction of reflection magnitude (RM; Table 2) is also consistent with a vascular adaptation as muscular arteries, which can change the magnitude or phase of wave reflection at various arterial interfaces. This is a key difference with arterial dysfunction preceding heart failure in humans, which is characterized by increased wave reflection increasing mid‐to‐late systolic load to the left ventricle (Chester et al., 2017; Chirinos, 2017; Chirinos et al., 2012; Zamani et al., 2016). Indeed, increased arterial stiffness and increased arterial wave reflection are considered components of the pathogenesis of the disease. Whether similar adaptations occur in humans with established heart failure and whether this differs in various types of heart failure remains to be determined.

Cardiac wave generation analyzed via WIA or WPA offers further assessment of hemodynamic function by identifying forward‐ and backward‐traveling wavefronts that are either of the compression or decompression (expansion) type (Broyd et al., 2017; Curtis et al., 2007; Mynard & Smolich, 2016; Weber & Chirinos, 2018). The initial forward compression wave (FCW, Figure 2) occurs at the onset of systole as blood is “pushed” from the LV to the aorta. The magnitude of FCW is associated with LV contraction; thus, decreased FCW height in our model suggests impaired LV contraction resulting from heart failure (Table 2; Figure 4D), which has also been observed in humans with heart failure (Curtis et al., 2007). In contrast, the height of the late‐systolic forward expansion wave (FEW) was unaffected by pacing in our study. The ratio between the heights of FCW and FEW was reduced with RVP and appears to represent a good indicator of ventricular systolic function, as it depends on the balance between compression and suction (Ntsinjana et al., 2017). The decreased ratio between accelerating and decelerating waves suggests a mechanism by which the myopathic heart expends relatively more energy decelerating blood flow and less energy accelerating it. This is likely an inefficient state of ventricular‐arterial interactions that may compromise pressure decay during isovolumic relaxation and the efficiency of diastolic suction. This remains to be determined in future studies.

WSA and WPA provide valuable diagnostic cardiac and vascular specific indices of CV function, but as both methods rely on established aortic pressure‐flow relationships, the aortic flow waveform is a critical component of any analyzed dataset. In humans, intermittent aortic flow is usually measured via Doppler echocardiography; in preclinical studies, including those designed to investigate test article‐dependent effects on heart and vascular function for safety or efficacy, aortic flow is best measured via surgically‐implanted aortic probes that enable continuous collection in conscious animals. This additional instrumentation limits situations where aortic flow can be collected. Therefore, reliable estimation of aortic flow via a preclinical synthetic flow algorithm offers an alternative approach for cases when true flow cannot be measured. In this study, a previously developed and validated synthetic flow algorithm was slightly adapted to estimate the proximal aortic flow profile (Shenouda et al., 2021). Directional changes from baseline at 1 week and 1 month measured with synthetic flow and true flow were in agreement for all but two indices (RM and wasted effort/QZc ratio at 1 month; Table 2), while the absolute value of indices were over or underestimated by relatively wide margins (Figure S1). The ratio between FCW and FEW heights was the only tested WPA index which was proven to be statistically identical in synthetic flow and measured flow cases at all three timepoints (Table 2). Further refinement of synthetic flow algorithms in dogs is possible and may better approximate the shape of the true aortic flow waveform and improve agreement between synthetic flow and measured flow‐derived WSA and WPA estimates.

It is important to note that these algorithms should not be used as a surrogate for direct preclinical CV assessment. Rather, the data presented support the conclusion that hemodynamic algorithms show value as supplementary tools in standard blood pressure assessment and could yield additional CV functional readouts that may prompt appropriate follow‐up, second tier assessments to potentially refine the risk assessment and/or to potentially improve the mechanistic understanding of the effects observed. Additionally, while the current study utilized central blood pressure waveforms collected in the thoracic aorta proximal to the aortic arch, it is appreciated that this is not the most common collection site for preclinical hemodynamic studies and use of more distal waveforms for such an approach would need to account for amplification and distortion of the waveform.

## 5 CONCLUSIONS

Taken together, these results show that preclinical hemodynamic algorithms such as PWA, WSA, and WPA can extract functional indices from canine arterial blood pressure and/or aortic flow that correlate with gold standards of cardiac function (SV) and/or expected hemodynamic outcomes in heart failure. The PWA index PSA was shown to be a reliable estimate of SV reduction induced by RVP. Additionally, WSA and WPA indices of pulsatility and wave reflection progressively reduced following RVP onset. Indices derived from synthesized flow waveforms exhibited similar directional changes to the same WSA and WPA indices derived from measured aortic flow. These results demonstrate the potential of pressure‐only or pressure‐flow analytical analyses of pulsatile hemodynamics to gain deeper insight into cardiovascular function and arterial hemodynamics in preclinical models, beyond standard blood pressure changes.

## FUNDING INFORMATION

This research did not receive any specific grant from funding agencies in the public, commercial, or not‐for‐profit sectors.

## CONFLICT OF INTEREST STATEMENT

Authors JH, TD, HR, and CR are employees of Merck Sharp & Dohme LLC, a subsidiary of Merck & Co., Inc., Rahway, NJ, USA.

## ETHICS STATEMENT

All animal studies were conducted in accordance with the Guide for the Care and Use of Laboratory Animals (Institute of Laboratory Animal Resources, Commission on Life Sciences, National Research Council, 2011) and were approved by the Institutional Animal Care and Use Committee at Merck & Co., Inc. (West Point, PA, USA).

## Supporting information


**Data S1.** Supporting information

**Figure S1.** Bland–Altman plots showing agreement between WSA and WPA indices derived using measured flow versus synthesized flow. The mean of the indices is plotted against the difference in the two measurements (measured flow – synthetic flow). Bias is indicated by a solid black line and the 95% limits of agreement are defined by the two dashed horizontal lines. Presented indices include: (A) QZc, (B) Wasted effort, (C) the ratio QZc/wasted effort, (D) forward wave amplitude (Pf), (E) backward wave amplitude (Pb), (G) reflection magnitude (RM = Pb/Pf), and (H) the ratio between forward compression wave (FCW) and forward expansion wave (FEW) heights.
**Figure S2**: Correlation plots for WSA and WPA indices derived using measured flow (X‐axis) versus synthesized flow (Y‐axis). The dashed line represents equality. Presented indices include: (A) QZc, (B) Wasted effort, (C) the ratio QZc/wasted effort, (D) forward wave amplitude (Pf), (E) backward wave amplitude (Pb), (G) reflection magnitude (RM = Pb/Pf), and (H) the ratio between forward compression wave (FCW) and forward expansion wave (FEW) heights.
