# Supplementary material for: Development and characterization of canine‐specific computational models to predict pulsatile arterial hemodynamics and ventricular‐arterial coupling
Source: Physiol Rep. 2023 Jun 3;11(11):e15731. doi: 10.14814/phy2.15731 (PMC10238860; doi:10.14814/phy2.15731)
Supplement: Supplementary file 2 — Figure S1. Bland–Altman plots showing agreement between WSA and WPA indices derived using measured flow versus synthesized flow. The mean of the indices is plotted against the difference in the two measurements (measured flow – synthetic flow). Bias is indicated by a solid black line and the 95% limits of agreement are defined by the two dashed horizontal lines. Presented indices include: (A) QZc, (B) Wasted effort, (C) the ratio QZc/wasted effort, (D) forward wave amplitude (Pf), (E) backward wave amplitude (Pb), (G) reflection magnitude (RM = Pb/Pf), and (H) the ratio between forward compression wave (FCW) and forward expansion wave (FEW) heights. Figure S2: Correlation plots for WSA and WPA indices derived using measured flow (X‐axis) versus synthesized flow (Y‐axis). The dashed line represents equality. Presented indices include: (A) QZc, (B) Wasted effort, (C) the ratio QZc/wasted effort, (D) forward wave amplitude (Pf), (E) backward wave amplitude (Pb), (G) reflection magnitude (RM = Pb/Pf), and (H) the ratio between forward compression wave (FCW) and forward expansion wave (FEW) heights. [file PHY2-11-e15731-s001.docx]

**Supplemental Material**


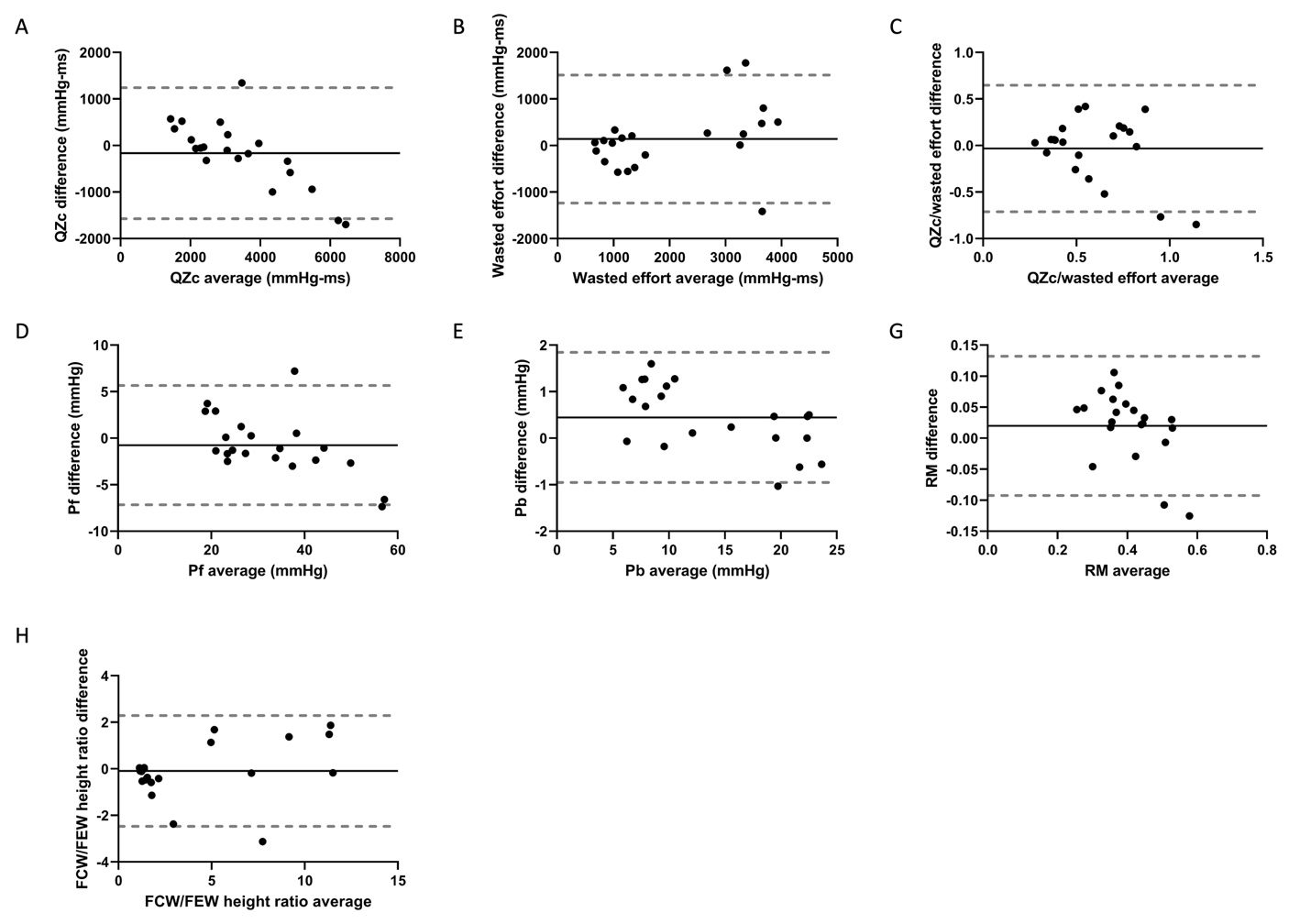


Figure S1: Bland-Altman plots showing agreement between WSA and WPA indices derived using measured flow vs. synthesized flow. The mean of the indices is plotted against the difference in the two measurements (measured flow – synthetic flow). Bias is indicated by a solid black line and the 95% limits of agreement are defined by the two dashed horizontal lines. Presented indices include: (A) QZc, (B) Wasted effort, (C) the ratio QZc/wasted effort, (D) forward wave amplitude (Pf), (E) backward wave amplitude (Pb), (G) reflection magnitude (RM = Pb/Pf), and (H) the ratio between forward compression wave (FCW) and forward expansion wave (FEW) heights.


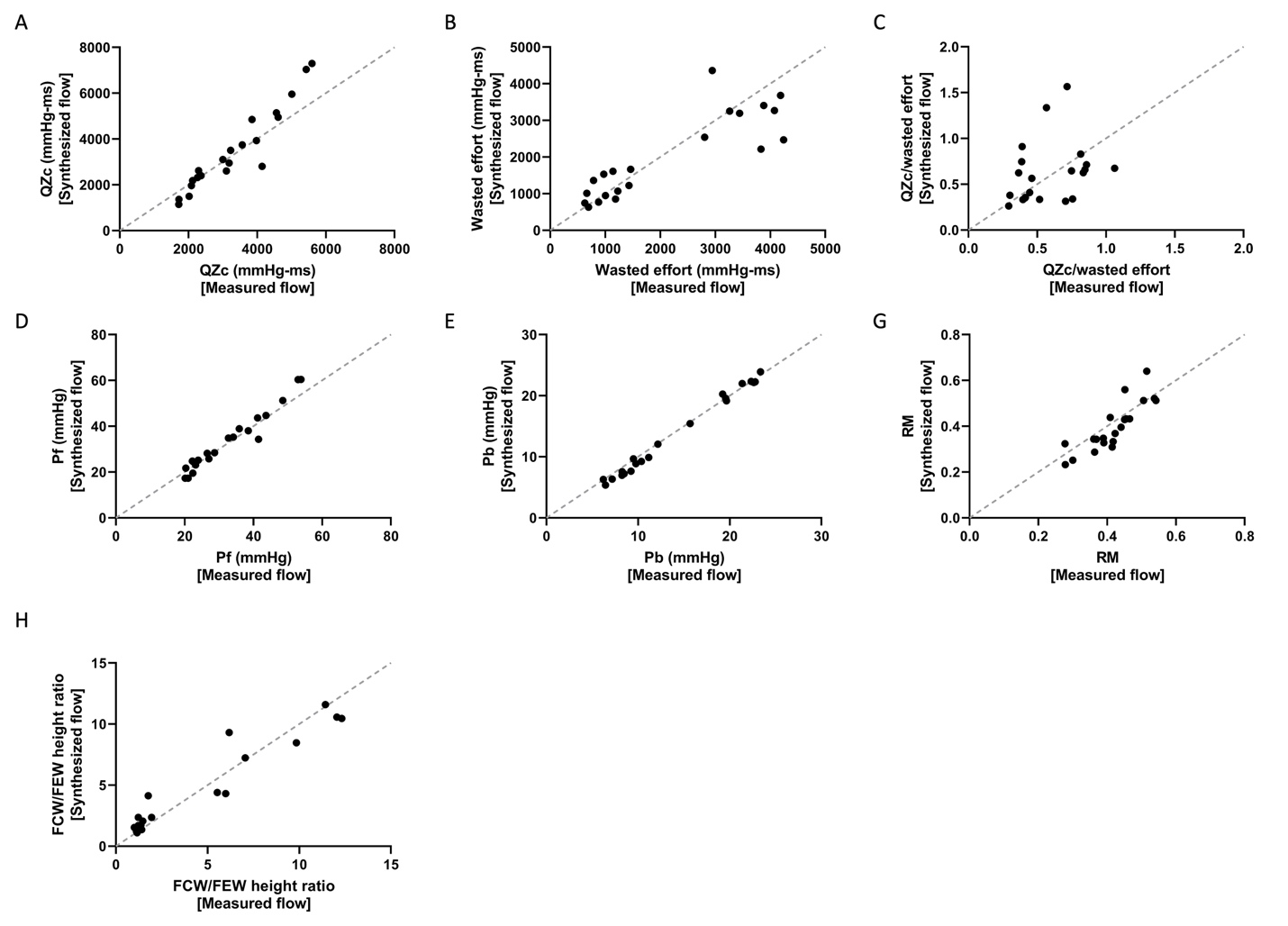


Figure S2: Correlation plots for WSA and WPA indices derived using measured flow (x-axis) vs. synthesized flow (y-axis). The dashed line represents equality. Presented indices include: (A) QZc, (B) Wasted effort, (C) the ratio QZc/wasted effort, (D) forward wave amplitude (Pf), (E) backward wave amplitude (Pb), (G) reflection magnitude (RM = Pb/Pf), and (H) the ratio between forward compression wave (FCW) and forward expansion wave (FEW) heights.
