# Supplementary material for: Development and characterization of canine‐specific computational models to predict pulsatile arterial hemodynamics and ventricular‐arterial coupling
Source: Physiol Rep. 2023 Jun 3;11(11):e15731. doi: 10.14814/phy2.15731 (PMC10238860; doi:10.14814/phy2.15731)
Supplement: Supplementary file 1 — Data S1. Supporting information [file PHY2-11-e15731-s002.pdf]

MERCK
770 Sumneytown Pike, WP81-1451
West Point, PA 19486 U.S.A.
C: +1 (610) 574-6018
E: julia.hotek@merck.com

5 April 2023

Dear Editor,

Enclosed please find our manuscript "Development and characterization of canine-specific computational models to predict pulsatile arterial hemodynamics and ventricular-arterial coupling" for publication in *Physiological Reports.*

In this work, we report on preclinical approaches of three common clinical computational tools for hemodynamic assessment: pulse wave analysis (PWA), wave separation analysis (WSA), and wave power analysis (WPA). These tools derive information about the ventricular-arterial system from blood pressure only or blood pressure-aortic flow pairs. While these tools have been used to characterize arterial hemodynamics in clinical datasets, there are very limited preclinical applications. We used a canine pacing-induced model of heart failure to characterize pulsatile hemodynamics with progression of heart failure using the preclinical algorithms, for the first time. We also evaluated the use of synthesized aortic flow waveforms derived from blood pressure by comparing WSA and WPA indices derived from measured flow to those derived from synthetic flow. Our data demonstrate the value of analytical hemodynamic tools to gain insight into preclinical cardiovascular effects using pressure-flow data or pressure alone. We anticipate this work having great impact especially in the preclinical space, as these tools add valuable complementary information to standard blood pressure endpoints commonly collected in safety and/or efficacy studies of new pharmaceutical agents.

We consider this work to be an excellent fit for the journal due to the scope of the manuscript and audience of the journal. This manuscript introduces the use of published clinical algorithms in a preclinical dataset for the first time and details modifications to these tools that were made to apply them to preclinical data. Further, the combination of experimental studies and computational tools to probe and model cardiovascular function will be of interest to the broad readership of the journal.

Thank you for taking your time to consider our manuscript. Please do not hesitate to contact us with any questions.

Sincerely,

Julia C. Hotek, PhD
